# Inhibitory effects of C-type natriuretic peptide on the differentiation of cardiac fibroblasts, and secretion of monocyte chemoattractant protein-1 and plasminogen activator inhibitor-1

**DOI:** 10.3892/mmr.2014.2763

**Published:** 2014-10-23

**Authors:** ZHI-QIANG LI, YING-LONG LIU, GANG LI, BIN LI, YANG LIU, XIAO-FENG LI, AI-JUN LIU

**Affiliations:** Department of Pediatric Cardiac Surgery Center, Beijing Anzhen Hospital, Capital Medical University, Beijing 100029, P.R. China

**Keywords:** cardiac fibroblast cell, cardiac fibrosis, C-type natriuretic peptide, differentiation, monocyte chemoattractant protein-1, plasminogen activator inhibitor-1

## Abstract

The present study aimed to investigate the effect of C-type natriuretic peptide (CNP) on the function of cardiac fibroblasts (CFs). Western blotting was used to investigate the expression of myofibroblast marker proteins: α-smooth muscle actin (α-SMA), extra domain-A fibronectin, collagen I and collagen III, and the activity of extracellular signal-regulated kinase 1/2 (ERK1/2). Immunofluorescence was used to examine the morphological changes; a transwell assay was used to analyze migration, and reverse transcription-quantitative polymerase chain reaction and ELISA were employed to determine the mRNA expression and protein secretion of monocyte chemoattractant protein-1 (MCP-1) and plasminogen activator inhibitor-1 (PAI-1). The results demonstrated that CNP significantly reduced the protein expression of α-SMA, fibronectin, collagen I and collagen III, and suppressed the migratory ability of CFs. Additionally, the mRNA and protein expression of MCP-1 and PAI-1 was inhibited under the CNP treatment; and this effect was mediated by the inhibition of the ERK1/2 activity. In conclusion, CNP inhibited cardiac fibroblast differentiation and migration, and reduced the secretion of MCP-1 and PAI-1, which demonstrates novel mechanisms to explain the antifibrotic effect of CNP.

## Introduction

Natriuretic peptides comprise a family of three structurally related peptides: Atrial natriuretic peptide (ANP), brain natriuretic peptide (BNP) and C-type natriuretic peptide (CNP). ANP and BNP are the primary cardiac hormones in the myocardium, which regulate cardiac remodeling through paracrine/autocrine actions. However, CNP, which was originally isolated from porcine brain extracts (1), not only has a regulatory effect in the central nervous system, but is also involved in the cardiovascular system (2). Besides the central nervous system and vascular endothelial cells, CNP is also synthesized by cardiac ventricular cells, mainly cardiac fibroblasts (CFs) in neonatal rats (3). Multifaceted cadioprotective effects of CNP have been previously reported (4–6), and it has been suggested that CNP may have more potent antifibrotic effects compared to ANP and BNP in cultured CFs (3).

Cardiac fibrosis is a significant aspect of cardiac remodeling, in which CFs are essential (7). Evidence reveals that CNP has a suppressive effect on cardiac fibrosis; however, the underlying cellular and molecular mechanisms have not been fully investigated. Whether and how CNP affects the function of CFs remains to be elucidated. Therefore, experiments were conducted in isolated and purified CFs in order to investigate whether CNP suppresses the differentiation of CFs into myofibroblasts and whether the secretion of profibrotic factors in CFs is affected by CNP. The present study demonstrated that CNP inhibits the differentiation of CFs into myofibroblasts *in vitro*, inhibits the migrational ability of CFs, and reduces the expression and secretion of monocyte chemoattractant protein-1 (MCP-1) and plasminogen activator inhibitor-1 (PAI-1) from CFs, which was mediated by the inhibition of the activity of extracellular signal-regulated kinase 1/2 (ERK1/2).

## Materials and methods

### Reagents

Dulbecco’s modified Eagle’s medium (DMEM), fetal bovine serum (FBS) and TRIzol reagent were purchased from Invitrogen Life Technologies (Carlsbad, CA, USA). CNP was purchased from Bachem (Torrance, CA, USA). Mouse monoclonal antibody against α-smooth muscle actin (α-SMA) was purchased from Sigma-Aldrich (St. Louis, MO, USA). Mouse monoclonal antibodies against glyceraldehyde 3-phosphate dehydrogenase (GAPDH), extra domain-A (ED-A) fibronectin, collagen I and III, and vimentin were purchased from Abcam (Cambridge, MA, USA). Mouse monoclonal antibodies against von Willebrand factor and troponin I were purchased from Santa Cruz Biotechnology, Inc. (Santa Cruz, CA, USA). Horseradish peroxidase (HRP)-conjugated, rhodamine-conjugated and fluorescein isothiocyanate (FITC)-conjugated secondary antibodies were obtained from BD Biosciences (San Jose, CA, USA). 4, 6-diamidino-2-phenylindole (DAPI) was purchased from Beyotime (Jiangsu, China). Antibodies against ERK1/2 and phospho (p)-ERK1/2 were purchased from Cell Signaling Technology, Inc. (Beverly, MA, USA). U0126 was purchased from Calbiochem (San Diego, CA, USA). An MCP-1 ELISA kit was obtained from R&D Systems (Minneapolis, MN, USA) and the PAI-1 ELISA kit was from American Diagnostica (Greenwich, CT, USA).

### Cell cultures

Primary cultures of neonatal rat CFs were prepared as described previously (8). Briefly, the hearts were excised from three-day-old Sprague-Dawley rats (n=46; Vital River Laboratory Animal Technology Co., Ltd., Beijing, China) and rinsed several times with phosphate-buffered saline. The ventricles were minced and trypsinized. Following centrifugation at 180 × g for 10 min, the cell pellets were resuspended. Floating cardiomyocytes and attached fibroblasts were separated. The purity of the cultured CFs was >95% on the basis of positive staining for vimentin and negative staining of troponin I and von Willebrand factor. Third-passage CFs were used in all the experiments. For subsequent experiments, cells at 80% confluence were growth-arrested by serum starvation for 24 h For myofibroblast differentiation and the cytokine secretion assay, CFs were cultured in DMEM with 10% FBS, with or without different concentrations of CNP (10^−9^, 10^−8^, 10^−7^ mol/l) for 24 h. In order to investigate the potential involvement of the ERK signaling pathway in the effects of CNP, CFs were pretreated with the pharmacological kinase inhibitor U0126 (10^−4^ mol/l) for 30 min, followed by treatment with CNP (10^−7^ mol/l). The cells and supernatants were harvested and stored at −80°C. All the procedures using animals were reviewed and approved by the Experimental Laboratory Animal Ethics Committee of Capital Medical University (Beijing, China) and were performed according to the criteria outlined by the National Ministry of Health.

### Western blot analysis

Western blot analysis was performed as previously described (9). Anti-α-SMA (1:1,000), anti-ED-A fibronectin (1:400), anti-collagen I (1:1,000), anti-collagen III (1:500), anti-ERK1/2 (1:2,000), anti-p-ERK1/2 (1:2,000) and anti-GAPDH (1:2,000) mouse monoclonal antibodies were used as the primary antibodies. The secondary antibody was HRP-conjugated anti-mouse IgG (1:4,000). The immunoreactive protein bands were detected using an enhanced chemiluminescence detection method (Applygen Technologies Inc., Beijing, China). Equal loading of the samples was further verified by staining of the blots with monoclonal antibodies against GAPDH. All the western blots were quantified using densitometry (Universal Hood; Bio-Rad Laboratories, Inc., Hercules, CA, USA).

### Immunofluorescence

CFs at passage 3 were seeded on coverslips and allowed to attach overnight. The cells were serum-starved for 24 h and subsequently treated with CNP for 24 h prior to staining with anti-α-SMA antibody (1:400) or anti-ED-A fibronectin antibody (1:200). Then cultures were incubated with Rhodamine- or FITC-conjugated secondary antibodies. Mounting medium containing DAPI was used to visualize the cell nuclei.

### Transwell migration assay

The ability of CFs to migrate across a matrix barrier towards chemotactic stimuli (in the present study 2% FBS was used) was investigated using the colorimetric transwell system QCM™ (Millipore Corporation, Billerica, MA, USA), which allows cells to migrate through a polycarbonate membrane with 8-μm pores. The CFs were serum starved for 24 h prior to the experiment. The cells were harvested and resuspended with serum-free DMEM, diluted to 5×10^5^ cells/ml. Subsequently, 300 μl cell suspensions were added to the upper chamber of each insert, 500 μl serum-free DMEM with 2% FBS was added to the lower chamber. CNP at the appropriate concentration was added to the upper and lower chambers of the experimental wells and incubation was at room temperature for 6 h. The migrated cells on the underside of the membrane were fixed by methanol treatment and stained with 0.1% crystal violet. Inserts were then dried and added to the extraction buffer. The optical density (OD) of the dye extract was measured at 560 nm using a microplate reader (Bio-Rad Laboratories, Inc.).

### Reverse transcription-quantitative polymerase chain reaction (RT-qPCR)

The total RNA was isolated using TRIzol reagent according to the manufacturer’s instructions. cDNA was generated with oligo (dT)_15_ primers (Promega Corporation, Madison, WI, USA). RT-qPCR was performed with Applied Biosystems 7500 real time PCR system using QuantiTect SYBR Green PCR Master mix (Applied Biosystems, Carlsbad, CA, USA). For normalization the housekeeping gene GAPDH was applied as a reference gene. The primer sequences are as follows: Forward: 5′-TGGCTCAGCCAGATGCAGT-3′ and reverse: 5′-ATTGGGATCATCTTGCTGGTG-3′ for MCP-1; PAI-1, forward: 5′-AACCCAGGCCGACTTCA-3′ and reverse: 5′-CATGCGGGCTGAGACTAGAAT-3′ for PAI-1; and forward: 5′-GGCAAATTCAACGGCACAGT-3′ and reverse: 5′-AGATGGTGATGGGCTTCCC-3′ for GAPDH. The comparative Ct (cycle threshold) method, also referred to as the 2^−ΔΔCT^ method, was used to quantify the gene expression. The relative gene expression was expressed as the ratio of CNP-treated to non-CNP-treated samples.

### ELISA assay

MCP-1 and PAI-1 concentrations in the supernatants were determined by ELISA kits, according to the manufacturer’s instructions. The OD of each well at 450 nm was measured using a microplate reader (Bio-Rad Laboratories, Inc.). The relative protein secretion was expressed as the ratio of CNP-treated to non-CNP-treated samples.

### Statistical analysis

The values are shown as the mean ± standard error of the mean. Analysis of variance and Student’s t-test were used for statistical analysis of the data. P<0.05 was considered to indicate a statistically significant difference.

## Results

### CNP inhibits myofibroblast differentiation of CFs

**Figure 1:**
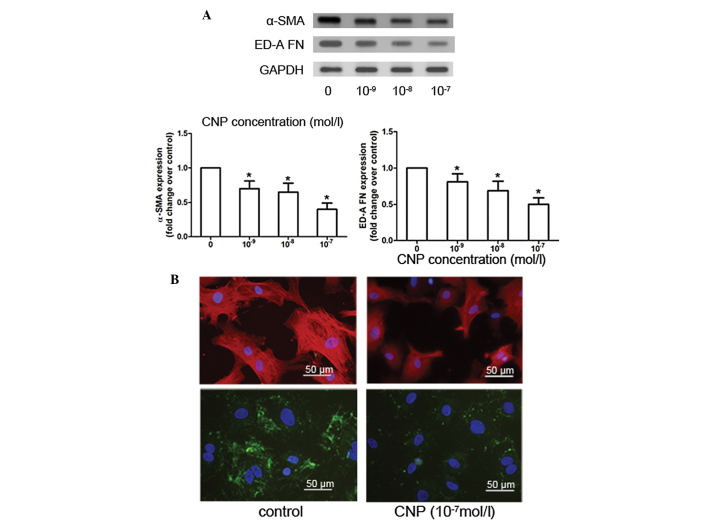
CNP inhibits α-SMA and ED-A fibronectin expression in CFs. (A) Western blot analysis and the corresponding densitometric quantification of α-SMA, ED-A fibronectin and GAPDH for culture after 24 h incubation with CNP concentrations of 10^−9^ to 10^−7^ mol/l. (B) α-SMA and ED-A FN immunofluorescent staining in CFs following treatment with 10^−7^ mol/l CNP. α-SMA and ED-A fibronectin were labeled with rhodamine (red) and FITC (green), and the nuclei were stained with DAPI (blue). Scale bar, 50 μm. ^*^P<0.05 compared with untreated CFs. CNP, C-type natriuretic peptide; α-SMA, α-smooth muscle actin; ED-A FN, extra domain-A fibronectin; CFs, cardiac fibroblasts; GAPDH, glyceraldehyde 3-phosphate dehydrogenase; DAPI, 4, 6-diamidino-2-phenylindole.

**Figure 2:**
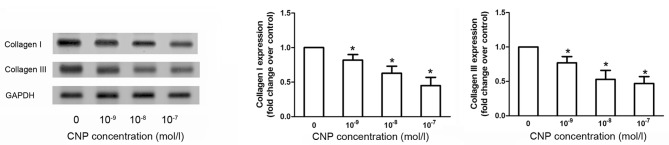
CNP inhibits collagen I and III expression in CFs. Western blot analysis and the corresponding densitometric quantification of collagen I and III, and GAPDH for culture after 24 h with CNP concentrations of 10^−9^ to 10^−7^ mol/l. ^*^P<0.05 compared with untreated CFs. CNP, C-type natriuretic peptide; GAPDH, glyceraldehyde 3-phosphate dehydrogenase.

To investigate the effect of CNP on myofibroblast differentiation of CFs, CFs isolated from neonatal rat ventricles were cultured in complete medium (DMEM with 10% FBS), and only third-passage CFs were analyzed. Prominent expression of α-SMA and ED-A fibronectin is the most well-characterized feature of myofibroblasts (10–14). In CFs treated with CNP, the protein expression of α-SMA and ED-A fibronectin was suppressed in a dose-dependent manner (Fig. 1A). Previous studies have shown that CFs cultured *in vitro* with DMEM containing FBS acquired a myofibroblast phenotype (13,15). Consistently, in the present study, it was revealed that CFs differentiated into myofibroblats with prominent stress fibers after 24 h in culture, demonstrated by the immunofluorescence staining of α-SMA, and the immunofluorescence signal was markedly reduced in the CNP-treated CFs. The inhibitory effect of CNP on the protein expression of ED-A fibronectin was also validated by immunofluorescence staining (Fig. 1B). In addition, western blot analysis results also revealed that CNP treatment decreased the expression of collagen I and III in CFs (Fig. 2).

### CNP inhibits cardiac fibroblast migration

**Figure 3:**
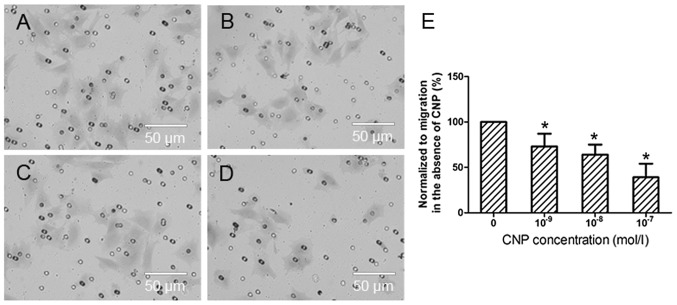
CNP inhibits cardiac fibroblast migration. A transwell migration assay was used to investigate the effects of CNP in CF migration. Representative images of (A) CNP-untreated and CNP-treated (B through D with a concentration of 10^−9^ to 10^−7^ mol/l, respectively) CFs that migrated toward 2% serum. (E) Quantitative analysis demonstrated that CNP-treated CFs revealed a reduced migratory capacity compared with untreated cells. Scale bar, 50 μm. ^*^P<0.05 compared with untreated CFs. CNP, C-type natriuretic peptide; CFs, cardiac fibroblasts.

The effects of CNP on cardiac fibroblast migration were examined by a transwell assay using 2% FBS as a chemotactic stimulus. The results demonstrated that treatment with CNP significantly reduced the number of migrating cells compared with the control group, as indicated by the cellular staining and the OD analysis of the cellular extraction (Fig. 3).

### CNP inhibits the expression and secretion of MCP-1 and PAI-1

**Figure 4:**
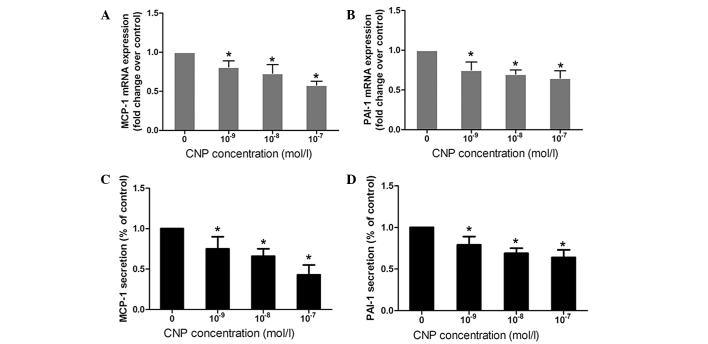
CNP inhibits the expression and secretion of MCP-1 and PAI-1. (A and B) MCP-1 mRNA expression determined by qPCR. (B) PAI-1 mRNA expression determined by qPCR. (C) MCP-1 protein secretion determined by ELISA. (D) PAI-1 protein secretion determined by ELISA. ^*^P<0.05 compared with untreated CFs. CNP, C-type natriuretic peptide; MCP-1, monocyte chemoattractant protein-1; PAI-1, plasminogen activator inhibitor-1.

RT-qPCR and ELISA assays were used to determine the effect of CNP on the RNA expression and protein secretion of MCP-1 and PAI-1 in CFs. RT-qPCR analyses demonstrated that treatment of CFs with CNP for 24 h resulted in a significant decrease in the mRNA expression of MCP-1 and PAI-1 (Fig. 4A and B). MCP-1 and PAI-1 protein concentrations in the culture medium of CNP-treated CFs were also significantly decreased compared with the control group (Fig. 4C and D).

### CNP inhibits activation of ERK1/2

**Figure 5:**
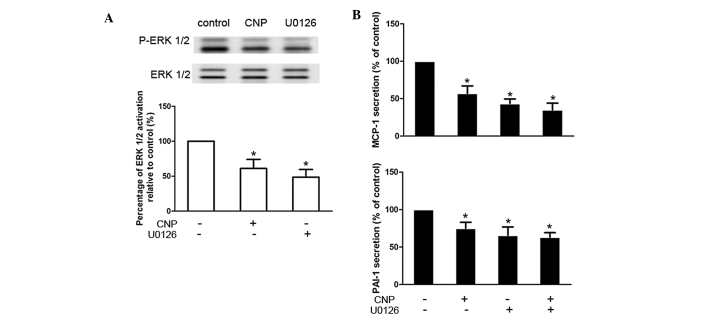
CNP inhibits the activation of ERK-MAPK. (A) Serum-starved cells were treated with CNP (10^−7^ mol/l) or pretreated with the ERK1/2 inhibitor U0126 (10^−4^ mol/l) for 30 min prior to culture with DMEM with 10% FBS for 30 min and then ERK1/2 phosphorylation was analyzed by western blot analysis. The signal was quantified by densitometry. (B) Serum-starved cells were treated with CNP (10^−7^ mol/l), or pretreated with the ERK1/2 inhibitor U0126 (10^−4^ mol/l) for 30 min prior to culture with DMEM with 10% FBS for 24 h, or pretreated with U0126 (10^−4^ mol/l) for 30 min prior to treatment with CNP (10^−7^ mol/l) for 24 h. MCP-1 and PAI-1 protein secretion in supernatants was determined by ELISA. ^*^P<0.05 compared with untreated CFs. CNP, C-type natriuretic peptide; ERK, extracellular signal-regulated kinase; MAP, mitogen activated protein; MCP, monocyte chemoattractant protein; PAI-1, plasminogen activator inhibitor-1; FBS, fetal bovine serum; DMEM, Dulbecco’s modified Eagle’s medium; CFs, cardiac fibroblasts.

In order to examine the influence of CNP on the downstream signaling pathways in CFs, experiments were conducted to analyze the effect of CNP on the activation of ERK1/2. The present study demonstrated that CNP effectively inhibited the protein expression of p-ERK1/2 (Fig. 5A). Additionally, the ELISA experiments revealed that the protein secretion of MCP-1 and PAI-1 was significantly decreased in the presence of the ERK1/2-specific inhibitor U0126 alone, and U0126 in combination with CNP (Fig. 5B).

## Discussion

Out of the three natriuretic peptides, CNP was the last to be identified (1). It has slowly emerged that CNP has a significant role in regulating cardiac function since its identification approximately two decades ago (16). The ability of CNP to protect against myocardial ischemia-reperfusion injury, and to inhibit cardiac fibrosis and hypertrophy has been reported (4–6). Additionally, elevated plasma CNP levels are identified in patients with heart failure (17,18).

CFs are fundamental to the normal structure of the heart and are essential during cardiac fibrosis, a pathological process that can ultimately result in heart failure. CFs are responsible for the deposition of the extracellular matrix and also secrete a number of inflammatory cytokines. Although previous studies have identified that CNP is a potent antifibrotic agent following myocardial infarction (5,19), the cellular mechanisms underlying this *in vivo* antifibrotic action of CNP are not fully understood. It has been reported that CNP inhibits proliferation and collagen synthesis of cultured CFs (3,5). However, the effects of CNP on other significant cardiac fibroblast functions, including differentiation, migration and cytokine secretion have not been investigated.

Cardiac myofibroblast differentiation is pivotal in the process of cardiac fibrosis. Following myocardial infarction, CFs migrate into the infarct border zone and differentiate into myofibroblasts promoting contraction of the scar (7,20). In the present study, it was revealed that CNP can inhibit the conversion of CFs to cardiac myofibroblasts, which was demonstrated by attenuated protein expression of α-SMA and fibronectin, which are key marker proteins of myofibroblast differentiation (10–14). This effect may confer beneficial antifibrotic effects. In addition, the expression of collagen I and III, which has also been demonstrated to be corerelated with the differentation of CFs into myofibroblasts (21–23), was investigated and results revealed that collagen I and III expression in CFs was inhibited following treatment with CNP. Cardiac fibroblast migration is another key process in cardiac fibrosis that usually accompanies differentiation (24). The *in vivo* migration was mimicked by a transwell assay, which enables CFs to traverse the matrigel-coated filters. The results demonstrated that CNP effectively inhibited the migration of CFs.

The inflammatory response, and cytokine secretion and production are particularly active following myocardial infarction (MI) and contribute to cardiac fibrosis and eventual cardiac dysfunction (25–27). MCP-1 is one of the most well-investigated cytokines in cardiac fibrosis (28). An elevated MCP-1 level was observed in the myocardium following MI and it has been demonstrated that MCP-1 has significant effects on macrophage recruitment and activation, cytokine synthesis and myofibroblast accumulation in healing infarcts (27,29–31). Disruption of the MCP-1 axis reduces fibrosis and attenuates dilation of the infarcted ventricle (32). With regard to PAI-1, a potent inhibitor of urokinase and tissue-type plasminogen activator, it is also critical in tissue fibrosis, including cardiac fibrosis (33). Evidence obtained in gene-deficient mice reveal that PAI-1 contributes to cardiac fibrosis (34,35), and it was reported that inhibition of PAI-1 is protective against the development of cardiac fibrosis (36). Therefore, as two profibrotic factors, MCP-1 and PAI-1 are key in the pathogenesis of cardiac fibrosis. In the present study, it was demonstrated that the mRNA expression and protein secretion of MCP-1 and PAI-1 in CFs were attenuated by CNP treatment, which may serve as another mechanism that underlies the anticardiac fibrotic properties of CNP.

The bioactivity of CNP is mainly mediated by its specific receptor NPR-B (2); however, the downstream signaling pathways have not been fully examined. In the present study, it was identified that CNP significantly decreased the activity of ERK1/2, a member of the mitogen-activated protein kinase (MAPK) superfamily, which was demonstrated to be involved in the regulation of various cytokines amongst numerous cell types (37). At the same time, the level of MCP-1 and PAI-1 was significantly decreased in the presence of U0126, an ERK1/2 inhibitor, which indicates that CNP inhibits MCP-1 and PAI-1 secretion via an ERK1/2-dependent mechanism.

In conclusion, CNP inhibited cardiac fibroblast differentiation and migration, and reduced MCP-1 and PAI-1 secretion in the CFs via the ERK1/2-MAPK signaling pathway, which implies novel mechanisms to explain the antifibrotic effect of CNP in the pathological process of cardiac remodeling.

## Abbreviations

CFs: cardiac fibroblasts
α-SMA: α-smooth muscle actin
ED-A FN: extra domain-A fibronectin
MCP-1: monocyte chemotattractant protein-1
PAI-1: plasminogen activator inhibitor-1
